# Controlling the Shannon Entropy of Quantum Systems

**DOI:** 10.1155/2013/381219

**Published:** 2013-05-30

**Authors:** Yifan Xing, Jun Wu

**Affiliations:** Institute of Cyber-Systems and Control, Zhejiang University, Hangzhou 310027, China

## Abstract

This paper proposes a new quantum control method which controls the Shannon entropy of quantum systems. For both discrete and continuous entropies, controller design methods are proposed based on probability density function control, which can drive the quantum state to any target state. To drive the entropy to any target at any prespecified time, another discretization method is proposed for the discrete entropy case, and the conditions under which the entropy can be increased or decreased are discussed. Simulations are done on both two- and three-dimensional quantum systems, where division and prediction are used to achieve more accurate tracking.

## 1. Introduction

Quantum control has become an important topic in quantum information [1, 2], molecular chemistry [3], and atomic physics [4]. Many control methods, including optimal control [5], Lyapunov control [6], learning control [7], feedback control [8], and incoherent control [9, 10], have been used in controller design of quantum systems. Our recent work [11, 12] has extended the classical probability density function (PDF) control method into quantum area. Based on classical PDF control, there is also a developing research area on Shannon entropy control, which has achieved good performance in classical systems, such as stochastic control [13, 14], networked control [15], and biological control [16]. The extension of Shannon entropy control into quantum area may also enhance quantum control performance. 

Shannon entropy in atomic calculations has further been related to various properties such as atomic ionization potential [17], molecular geometric parameters [18], chemical similarity of different functional groups [19], characteristics of correlation methods for global delocalizations [20], molecular reaction paths [21], orbital-based kinetic theory [22], highly excited states of single-particle systems [23], and nature of chemical bonds [24]. The consistency of the Shannon entropy when applied to outcomes of quantum experiments has been analyzed [25], and it is shown that Shannon entropy is fully consistent and its properties are never violated in quantum settings.

In the recent research about quantum sliding-mode control (SMC) [26, 27], a sliding mode is defined based on the fidelity with a desired eigenstate, and the goal is to maintain the state in the mode or drive it back into the mode after measurement. In fact, the fidelity here is directly related to Shannon entropy. There is also research about coherent control based on tracking control for two-level systems [28]. Since coherence corresponds to large entropy, while fidelity corresponds to small entropy, we can directly control the entropy to achieve the goal. If the entropy can track a desired trajectory, the state will be able to slide among different modes, rather than in one mode in the existing quantum SMC. For *n*-level systems which cannot be depicted by Bloch sphere, such method can also provide a systematic way to maintain fidelity or coherence.

For the biological and physiological datasets, quantifying disorder of the system has become popular as an intense area of promising recent research. In the recent study of a complexity measure for nonstationary signals [16], Shannon entropy has been used to distinguish “healthy” from “unhealthy” biological signals. The study has quantified the information evolution of transitions associated with probabilities assigned to each state, with a goal of providing single value (an entropy) to describe the information content. Similar approach can be adopted to systems where the change in parameter would be indicative of a change in the “health” of the system. For example, in the recent research about information theoretic measures of the electron correlation for both continuous [29] and discrete [30] cases, it is shown that Shannon entropy can also provide a new way to calculate electron correlation energy more accurately. An accurate description of atomic and molecular properties requires an explicit account of electron correlation, while there is no operator in quantum mechanics whose measurement gives the correlation energy. Since strong correlation corresponds to large entropy, we can also use Shannon entropy as a new approach to control quantum correlation.

Quantum von Neumann entropy is a good measure of entanglement, and it will reduce to Shannon entropy for the pure state case. It can provide a real-time noise observation and a systematic guideline to make reasonable choice of control strategy. The von Neumann entropy is just a measure of the purity of the given density matrix without explicit reference to information contained in individual measurements [31]. While quantum Shannon entropy can reveal a great deal of information from the perspective of geometrical changes to the density [21], it shows interesting features about the bond forming and breaking process that are not apparent from the conventional reaction energy profile. Recent research has studied how to image and manipulate the shape of electronic wavefunction [32] and how to directly measure the quantum wavefunction for photons [33]. If the probability density function can be well measured and controlled in the future, we can directly control the detailed spatial distribution for both pure and mixed states. Sometimes, the detailed distribution may not be important, while we only need to make the distribution more ordered or disordered. This also calls for the control of the uncertainty, which can be directly reflected by Shannon entropy.

This paper provides two primary methods to steer the discrete and continuous quantum Shannon entropy via quantum PDF control. And for the discrete case, a method based on discretization approximation is provided which can directly control the entropy and achieve more accurate performance. This paper is organized as follows.  Section 2 presents the basic quantum control model and the definitions of both discrete and continuous quantum Shannon entropy. Sections 3 and 4 provide the controller design methods based on PDF control for discrete and continuous entropy, respectively.  Section 5 provides a direct control method for discrete entropy based on discretization approximation.  Section 6 shows the numerical simulation examples. Concluding remarks are given in Section 7.

## 2. Preliminary

In quantum control, the state of a closed quantum system is represented by a state vector (wavefunction) *ψ*(*x*, *t*) in a Hilbert space. Here, for the space variable we only consider one-dimensional position variable *x*. The evolution of the state obeys the Schrödinger equation:

(1) $$\begin{array}{c} \iota \hbar \dot{\psi }\left(x,t\right)=\left[-\frac{\hbar^{2}}{2m}\cdot \frac{\partial^{2}}{\partial x^{2}}+U\left(x,t\right)\right]\psi \left(x,t\right), \end{array}$$

where $\iota =\sqrt{-1}$ and the external potential field *U*(*x*, *t*) ∈ **R** is taken as the control term. For an infinite dimensional quantum system, the wavefunction *ψ*(*x*, *t*) is the superposition of free Hamiltonian's eigenstates *ψ*_*i*_(*x*):

(2) $$\begin{array}{c} \psi \left(x,t\right)=\sum_{i=1}^{\infty }c_{i}\left(t\right)\psi_{i}\left(x\right), \end{array}$$

where both the wavefunction and the coefficients should be normalized:

(3) $$\begin{array}{c} \int_{-\infty }^{\infty }{\left|\psi \left(x,t\right)\right|}^{2}\text{d}x=\sum_{i=1}^{\infty }{\left|c_{i}\left(t\right)\right|}^{2}=1. \end{array}$$

Defining the state of the system as follows:

(4) $$\begin{array}{c} C\left(t\right)={\left[c_{1}\left(t\right),c_{2}\left(t\right),\ldots ,c_{n}\left(t\right),\ldots \right]}^{T}, \end{array}$$

and the Schrödinger equation can be written as follows:

(5) $$\begin{array}{c} \dot{C}\left(t\right)=\left[A+\sum_{i=1}^{k}B_{i}U_{i}\left(t\right)\right]C\left(t\right), \end{array}$$

where both *A* and *B*_*i*_ are skew-Hermitian matrices. If the case with only one control *U*(*t*) can be well solved, it will be easier for multiple control cases. So, this paper only considers the following case with one control:

(6) $$\begin{array}{c} \dot{C}\left(t\right)=AC\left(t\right)+BU\left(t\right)C\left(t\right). \end{array}$$

Assuming a system that consists of *n* states, in which the probability for the *i*th state to happen is *p*_*i*_, the traditional discrete Shannon entropy in information science is defined as follows:

(7) $$\begin{array}{c} S_{d}=-\sum_{i=1}^{n}p_{i}\ln p_{i}, \end{array}$$

which shows the degree of randomness of the system. For example, when *p*_1_ = *p*_2_ = ⋯ = *p*_*n*_ = 1/*n*, every state happens in the equal probability, which is a random system. In this situation, the Shannon entropy takes its maximum value ln⁡*n*. If *p*_1_ = 1, the system is completely predictable; that is, the first state always happens, and the entropy takes its minimum value 0. We can also regard the entropy as the superposition of the uncertainties ln⁡(1/*p*_*i*_) because larger probability can lead to smaller uncertainty. Similarly, the discrete quantum Shannon entropy can be defined as follows:

(8) $$\begin{array}{c} S_{d}\left(t\right)=-\sum_{i=1}^{\infty }{\left|c_{i}\left(t\right)\right|}^{2}\ln{\left|c_{i}\left(t\right)\right|}^{2}, \end{array}$$

where |*c*_*i*_(*t*)|^2^ is the probability that the superposition state collapses to the *i*th eigenstate upon quantum measurement. Next, for the continuous case, Shannon proposed that the entropy for a system with a probability distribution *p*(*x*) in one dimension could be characterized by the following:

(9) $$\begin{array}{c} S_{c}=-\int p\left(x\right)\ln p\left(x\right)\text{d}x,\;\;\int p\left(x\right)\text{d}x=1, \end{array}$$

which measures the delocalization or the lack of structure in the respective distribution. Thus, the entropy is maximal for uniform distribution and is minimal when the uncertainty about the structure of the distribution is minimal. Since the quantum probability density can be denoted by a continuous function |*ψ*(*x*,*t*)|^2^, we can define continuous quantum Shannon entropy as follows:

(10) $$\begin{array}{c} S_{c}\left(t\right)=-\int_{-\infty }^{\infty }{\left|\psi \left(x,t\right)\right|}^{2}\ln{\left|\psi \left(x,t\right)\right|}^{2}\text{d}x, \end{array}$$

where integral can be used to deal with continuous probability distribution. Our goal is to drive the entropy from any initial value to any target.

## 3. Controller Design for Discrete Entropy Based on PDF Control

Here, we consider finite dimensional quantum systems. From definition (8), we know that the discrete entropy satisfies the following:

(11) $$\begin{array}{c} S_{d}\left(t\right)=-\sum_{i=1}^{n}{\left|c_{i}\left(t\right)\right|}^{2}\ln{\left|c_{i}\left(t\right)\right|}^{2}\geq 0. \end{array}$$

It is clear that when |*c*_1_(*t*)|^2^ = |*c*_2_(*t*)|^2^ = ⋯ = |*c*_*n*_(*t*)|^2^ = 1/*n*, *S*_*d*_(*t*) reaches its maximum ln⁡*n*. *S*_*d*_(*t*) reaches its minimum 0 when

(12) $$\begin{array}{c} {\left|c_{i}\left(t\right)\right|}^{2}=\{\begin{array}{cc} 1, & i=k,\\ 0, & i\neq k, \end{array} \end{array}$$

where *k* is a given integer. This conclusion can be proved using the following fact:

(13) $$\begin{array}{c} \underset{x\to 0}{\lim}x\ln x=\underset{x\to 0}{\lim}\frac{\ln x}{1/x}=\underset{x\to 0}{\lim}\frac{\left(\text{d}/\text{d}x\right)\left(\ln x\right)}{\left(\text{d}/\text{d}x\right)\left(1/x\right)}=\underset{x\to 0}{\lim}\left(-x\right)=0. \end{array}$$

The control of *S*_*d*_(*t*) can be realized by controlling the probability density |*c*_*i*_(*t*)|^2^. 

Denote the target of *C*(*t*) as follows:

(14) $$\begin{array}{c} C={\left[c_{1},c_{2},\ldots ,c_{n}\right]}^{T}, \end{array}$$

which satisfies the normalization condition

(15) $$\begin{array}{c} \sum_{i=1}^{n}{\left|c_{i}\right|}^{2}=1. \end{array}$$

There are several methods [6, 34] to reach the target under some assumptions, though the asymptotic stability may not be guaranteed. Here, we provide another method which can deal with any final condition without guaranteeing the asymptotic stability. First, we define the error as follows:

(16) $$\begin{array}{c} e\left(t\right)=\sum_{i=1}^{n}{\left|c_{i}\left(t\right)-c_{i}\right|}^{2}. \end{array}$$

In order to make the error decrease, we let

(17) $$\begin{array}{c} \dot{e}\left(t\right)=-ke\left(t\right), \end{array}$$

where *k* ∈ **R** ^+^can be preselected. Substituting (3), (14), and (15) into (16) we have the following:

(18) e(t)=∑i=1n[ci(t)−ci]∗[ci(t)−ci]=∑i=1n|ci(t)|2+∑i=1n|ci|2−∑i=1n[ci∗ci(t)+ci∗(t)ci]=2−2∑i=1nℜ[ci∗ci(t)]=2−2ℜ[∑i=1nci∗ci(t)]=2−2ℜ[C+C(t)],

where *C* ^+^ = (C*)^*T*^. Then we can obtain the following relationship based on (6), (17), and (18):

(19) e˙(t)=−2ℜ[C+C˙(t)]=−2ℜ{C+[A+U(t)B]C(t)}=−2ℜ[C+AC(t)]−2U(t)ℜ[C+BC(t)]=−k{2−2ℜ[C+C(t)]}.

From (19), we can get the following controller:

(20) $$\begin{array}{c} U\left(t\right)=\frac{k-ℜ\left\{C^{+}\left[A+kI\right]C\left(t\right)\right\}}{ℜ\left[C^{+}BC\left(t\right)\right]}. \end{array}$$

This is the desired controller which can make the error decrease.

 When the state has reached its target, in order to keep it unchanged, we can do the following calculation about the derivative of the probability density:

(21) ddt[|c1(t)|2|c2(t)|2⋮|cn(t)|2] =ddt[C(t)∘C∗(t)]=C˙(t)∘C∗(t)+C(t)∘C˙∗(t) =2ℜ[C˙(t)∘C∗(t)] =2ℜ{[AC(t)+BU(t)C(t)]∘C∗(t)} =2ℜ[AC(t)∘C∗(t)]+2U(t)ℜ[BC(t)∘C∗(t)],

where “∘” is defined as follows, which means the corresponding elements are multiplied:

(22) $$\begin{array}{c} \left[\begin{array}{c} a_{1}\\ a_{2}\\ \vdots \\ a_{n} \end{array}\right]\circ \left[\begin{array}{c} b_{1}\\ b_{2}\\ \vdots \\ b_{n} \end{array}\right]=\left[\begin{array}{c} a_{1}b_{1}\\ a_{2}b_{2}\\ \vdots \\ a_{n}b_{n} \end{array}\right]. \end{array}$$

From (21), it is easy to find that in order to keep the probability constant, we only need the following:

(23) $$\begin{array}{c} ℜ\left[AC\left(t\right)\circ C^{*}\left(t\right)\right]+U\left(t\right)ℜ\left[BC\left(t\right)\circ C^{*}\left(t\right)\right]\equiv {\left[\begin{array}{c} 0\\ 0\\ \vdots \\ 0 \end{array}\right]}_{n\times 1}. \end{array}$$

When *A* is diagonal, all the elements in *A* should be pure imaginary because *A* is skew-Hermitian. Assuming *A* = diag⁡{*a*_11_ *ι*, *a*_22_ *ι*,…, *a*_*nn*_ *ι*}  (*a*_*ii*_ ∈ **R**), we have the following:

(24) ℜ[AC(t)∘C∗(t)] =ℜ{ι[a11|c1(t)|2+a22|c2(t)|2+⋯+ann|cn(t)|2]}=0.

Hence, once the system's entropy reaches the target, we can use *U*(*t*) = 0 to maintain the entropy unchanged. For other quantum systems with nondiagonal *A*, it is not easy to keep the entropy unchanged with (23). Then we will develop an approximation method in Section 5 to achieve good performance.

## 4. Controller Design for Continuous Entropy Based on PDF Control

From definition (10), we know *S*_*c*_(*t*) ≥ 0. It is easy to prove that *S*_*c*_(*t*) can reach its maximum when the probability distribution |*ψ*(*x*,*t*)|^2^ is a uniform distribution and can reach its minimum when the uncertainty about the structure of the distribution is minimal, for example, a delta-like distribution. We can control the continuous entropy by controlling |*ψ*(*x*,*t*)|^2^.

Define the target distribution of *ψ*(*x*, *t*) as *ψ*_*d*_(*x*) which satisfies the following:

(25) $$\begin{array}{c} \int_{-\infty }^{\infty }{\left|\psi_{d}\left(x\right)\right|}^{2}\text{d}x=1. \end{array}$$

The error can be defined as follows:

(26) $$\begin{array}{c} e\left(t\right)=\int_{-\infty }^{\infty }{\left|\psi \left(x,t\right)-\psi_{d}\left(x\right)\right|}^{2}\text{d}x. \end{array}$$

The goal is to make the error decrease in this way:

(27) $$\begin{array}{c} \dot{e}\left(t\right)=-ke\left(t\right). \end{array}$$

Based on (3) and (25), we can rewrite *e*(*t*) as follows:

(28) e(t)=∫−∞∞[ψ(x,t)−ψd(x)]∗[ψ(x,t)−ψd(x)]dx=∫−∞∞|ψ(x,t)|2dx+∫−∞∞|ψd(x)|2dx−∫−∞∞[ψ∗(x,t)ψd(x)+ψd∗(x)ψ(x,t)]dx=2−2∫−∞∞ℜ[ψd∗(x)ψ(x,t)]dx.

Then we can obtain the following relationship based on (1), (27), and (28):

(29) e˙(t) =−2∫−∞∞ℜ[ψd∗(x)ψ˙(x,t)]dx =−2∫−∞∞ℜ{ψd∗(x)        ×[ιℏ2m·∂2ψ(x,t)∂x2−ιℏU(t)ψ(x,t)]}dx =−ℏm∫−∞∞ℜ[ιψd∗(x)∂2ψ(x,t)∂x2]dx  +2U(t)ℏ∫−∞∞ℜ[ιψd∗(x)ψ(x,t)]dx =ℏm∫−∞∞ℑ[ψd∗(x)∂2ψ(x,t)∂x2]dx  −2U(t)ℏ∫−∞∞ℑ[ψd∗(x)ψ(x,t)]dx =−k{2−2∫−∞∞ℜ[ψd∗(x)ψ(x,t)]dx}.

From (29), we can get the following controller:

(30) U(t)=(ℏ22m∫−∞∞ℑ[ψd∗(x)∂2ψ(x,t)∂x2]dx −kℏ∫−∞∞ℜ[ψd∗(x)ψ(x,t)]dx+kℏ)×(∫−∞∞ℑ[ψd∗(x)ψ(x,t)]dx)−1.

This is the desired controller which can make the error decrease when applied to the quantum system. We can substitute (30) into the Schrödinger equation (1) to solve *U*(*t*) out because only *ψ*(*x*, *t*) and *U*(*t*) are unknown. This task can be numerically accomplished by computer simulation or discretization. Moreover, in practice, some methods have been developed for the real-time measurement of quantum PDF under some special cases [32, 35]. If the quantum PDF can be measured online in the future, we can directly measure *ψ*(*x*, *t*) and calculate *U*(*t*) with (30).

When *ψ*(*x*, *t*) → *ψ*_*d*_(*x*), *U*(*t*) will not be asymptotic stable. We can design an external field to make *ψ*(*x*, *t*) unchanged when it is near to *ψ*_*d*_(*x*) at time *t*_*f*_. To make *ψ*(*x*, *t*) unchanged is just to make

(31) $$\begin{array}{c} \forall t>t_{f},\;\psi \left(x,t\right)=\psi \left(x,t_{f}\right). \end{array}$$

Substituting (31) into the Schrödinger equation (1) we obtain the following:

(32) $$\begin{array}{c} 0=-\frac{\hbar^{2}}{2m}\cdot \frac{{\text{d}}^{2}\psi \left(x,t_{f}\right)}{\text{d}x^{2}}+U\left(x,t\right)\psi \left(x,t_{f}\right), \end{array}$$

which gives the following:

(33) $$\begin{array}{c} U\left(x,t\right)=U\left(x\right)=\frac{\hbar^{2}}{2m\cdot \psi \left(x,t_{f}\right)}\cdot \frac{{\text{d}}^{2}\psi \left(x,t_{f}\right)}{\text{d}x^{2}}. \end{array}$$

Such a field will keep *ψ*(*x*, *t*) constant.

## 5. Controller Design for Discrete Entropy Based on Discretization Approximation

In the above two methods, the entropy does not truly enter the control procedure and cannot be driven to the target at any prespecified time. To achieve more direct and accurate control, we can adopt discretization to clarify the relationship between the entropy and the controller. 

Assuming the sampling period is *T*, the control model (6) with dimension *n* can be descretized as follows:

(34) $$\begin{array}{c} \frac{C\left(T\right)-C\left(0\right)}{T}=AC\left(0\right)+BU\left(0\right)C\left(0\right), \end{array}$$

where *C*(0) is the initial state, *C*(*T*) is the state at time *T*, and *U*(0) is the external potential field which will remain constant in the first sampling period *T*. Then we have the following:

(35) $$\begin{array}{c} C\left(T\right)=\left(I+TA\right)C\left(0\right)+TBU\left(0\right)C\left(0\right), \end{array}$$

where *I* is the identity matrix with dimension *n*. For finite dimensional quantum systems, the derivative of the discrete entropy (8) is as follows:

(36) dSd(t)dt=−∑i=1n[ln⁡|ci(t)|2+1]d|ci(t)|2dt=−∑i=1nd|ci(t)|2dtln⁡|ci(t)|2−∑i=1nd|ci(t)|2dt.

It is clear that

(37) $$\begin{array}{c} \sum_{i=1}^{n}\frac{\text{d}{\left|c_{i}\left(t\right)\right|}^{2}}{\text{d}t}=\frac{\text{d}}{\text{d}t}\left[\sum_{i=1}^{n}{\left|c_{i}\left(t\right)\right|}^{2}\right]\equiv 0. \end{array}$$

Hence (36) can be changed into the following:

(38) $$\begin{array}{c} \frac{\text{d}S_{d}\left(t\right)}{\text{d}t}=-\sum_{i=1}^{n}\frac{\text{d}{\left|c_{i}\left(t\right)\right|}^{2}}{\text{d}t}\ln{\left|c_{i}\left(t\right)\right|}^{2}. \end{array}$$

We discretize (38) as

(39) $$\begin{array}{c} \frac{S_{d}\left(T\right)-S_{d}\left(0\right)}{T}=-\sum_{i=1}^{n}\frac{{\left|c_{i}\left(t\right)\right|}^{2}-{\left|c_{i}\left(0\right)\right|}^{2}}{T}\ln{\left|c_{i}\left(0\right)\right|}^{2}, \end{array}$$

which implies

(40) Sd(T)−Sd(0) =−∑i=1n|ci(t)|2ln⁡|ci(0)|2+∑i=1n|ci(0)|2ln⁡|ci(0)|2.

It is clear that

(41) $$\begin{array}{c} S_{d}\left(0\right)=-\sum_{i=1}^{n}{\left|c_{i}(0)\right|}^{2}\ln{\left|c_{i}(0)\right|}^{2}. \end{array}$$

Substituting (41) into (40) leads to the following:

(42) $$\begin{array}{c} S_{d}\left(T\right)=-\sum_{i=1}^{n}{\left|c_{i}\left(T\right)\right|}^{2}\ln{\left|c_{i}\left(0\right)\right|}^{2}. \end{array}$$

Here, we use −∑_*i*=1_^*n*^|*c*_*i*_(*T*)|^2^ln⁡|*c*_*i*_(0)|^2^ to approximate −∑_*i*=1_^*n*^|*c*_*i*_(*T*)|^2^ln⁡|*c*_*i*_(*T*)|^2^. The following theorem shows that not only the approximation is feasible, but also the approximation error is an infinitesimal of higher order than the change of probability under small change of the probability.

**Theorem 1**

When |*c*_*i*_(0)|^2^ ≠ 0  (for all  *i*) holds, if the probability change is very small (for all  *i*, |*c*_*i*_(*T*)|^2^ − |*c*_*i*_(0)|^2^ → 0), *S*_*d*_(*T*) = −∑_*i*=1_^*n*^|*c*_*i*_(*T*)|^2^ln⁡|*c*_*i*_(*T*)|^2^  (*T* → 0) can be approximated by −∑_*i*=1_^*n*^|*c*_*i*_(*T*)|^2^ln⁡|*c*_*i*_(0)|^2^, and the approximation error *e* = ∑_*i*=1_^*n*^|*c*_*i*_(*T*)|^2^ln⁡|*c*_*i*_(*T*)|^2^ − ∑_*i*=1_^*n*^|*c*_*i*_(*T*)|^2^ln⁡|*c*_*i*_(0)|^2^ is an infinitesimal of higher order than the probability change ∑_*i*=1_^*n*^||*c*_*i*_(*T*)|^2^ − |*c*_*i*_(0)|^2^|.

**Proof**

Assume |*c*_*i*_(0)|^2^ = *p*_*i*_ ≠ 0, |*c*_*i*_(*T*)|^2^ − |*c*_*i*_(0)|^2^ = Δ_*i*_, and *e* can be written as follows:

(43) e=∑i=1n(pi+Δi)ln⁡(pi+Δi)−∑i=1n(pi+Δi)ln⁡pi=∑i=1n(pi+Δi)ln⁡pi+Δipi.

It is clear that

(44) lim⁡Δi→0(pi+Δi)ln⁡((pi+Δi)/pi)Δi =lim⁡Δi→0ln⁡((pi+Δi)/pi)Δi/(pi+Δi) =lim⁡Δi→0(d/dΔi)(ln⁡((pi+Δi)/pi))(d/dΔi)(Δi/(pi+Δi)) =lim⁡Δi→0(pi/(pi+Δi))·(1/pi)(pi+Δi−Δi)/(pi+Δi)2 =lim⁡Δi→0(1+Δi/pi)=1,

so we can get lim_Δ_*i*_→0_(*p*_*i*_ + Δ_*i*_)ln⁡((*p*_*i*_ + Δ_*i*_)/*p*_*i*_) = lim_Δ_*i*_→0_Δ_*i*_, which implies lim_Δ_*i*_→0_ *e* = lim_Δ_*i*_→0_∑_*i*=1_^*n*^Δ_*i*_ = 0. Since the limit of the approximation error is zero, we can say the approximation is feasible. Moreover, we have the following:

(45) $$\begin{array}{c} \underset{\Delta_{i}\to 0}{\lim}\frac{e}{\sum_{i=1}^{n}\left|{\left|c_{i}\left(T\right)\right|}^{2}-{\left|c_{i}\left(0\right)\right|}^{2}\right|}=\underset{\Delta_{i}\to 0}{\lim}\frac{\sum_{i=1}^{n}\Delta_{i}}{\sum_{i=1}^{n}\left|\Delta_{i}\right|}=0. \end{array}$$

Hence,

(46) $$\begin{array}{c} e=o\left(\sum_{i=1}^{n}\left|{\left|c_{i}\left(T\right)\right|}^{2}-{\left|c_{i}\left(0\right)\right|}^{2}\right|\right). \end{array}$$

Theorem 1 allows us to use (42) to approximate the entropy after change. Here, for simplicity we define a row vector:

(47) D≜[−ln⁡|c1(0)|2,−ln⁡⁡|c2(0)|2,…,ln⁡⁡|cn(0)|2]=[d1,d2,…,dn]∈R1×n.

Since 0 ≤ |*c*_*i*_(0)|^2^ ≤ 1, we know *d*_*i*_ ≥ 0. Substituting (47) into (42) leads to the following:

(48) Sd(T)=D[C(T)∘C∗(T)]=D{[(I+TA)C(0)+U(0)TBC(0)]  ∘[(I+TA∗)C∗(0)+U(0)TB∗C∗(0)]}=D{(I+TA)C(0)∘(I+TA∗)C∗(0)  +U2(0)TBC(0)∘TB∗C∗(0)  +U(0)[TBC(0)∘(I+TA∗)C∗(0)      +(I+TA)C(0)∘TB∗C∗(0)]}.

Define

(49) M≜TBC(0)∘TB∗C∗(0)∈Rn×1,N≜TBC(0)∘(I+TA∗)C∗(0)+(I+TA)C(0)∘TB∗C∗(0)∈Rn×1,K≜(I+TA)C(0)∘(I+TA∗)C∗(0)∈Rn×1,

which can change (48) into the following:

(50) $$\begin{array}{c} S_{d}\left(T\right)=U^{2}\left(0\right)DM+U\left(0\right)DN+DK. \end{array}$$

It is clear that all the elements in *M* and *K* are nonnegative, which lead to *DM* ≥ 0 and *DK* ≥ 0. When *DM* = 0, to make the entropy in (50) reach its target, we can simply choose *U*(0) = (*S*_*d*_(*T*) − *DK*)/*DN*. But in most cases we have *DM* > 0, and from (50) the one-step controller can be calculated as follows:

(51) $$\begin{array}{c} U\left(0\right)=\frac{-DN\pm \sqrt{{\left(DN\right)}^{2}-4DM\left[DK-S_{d}\left(T\right)\right]}}{2DM}. \end{array}$$

Here, the selection of plus and minus depends on the value of |*U*(0)|, and detailed discussions can be found in Section 6.1. Since *U*(0) belongs to the real domain, we have (*DN*)^2^ − 4*DM*[*DK* − *S*_*d*_(*T*)] ≥ 0, which leads to the following:

(52) $$\begin{array}{c} S_{d}\left(T\right)\geq DK-\frac{{\left(DN\right)}^{2}}{4DM}. \end{array}$$

This means that *S*_*d*_(*T*) has a lower bound.  Proposition 2 shows that the lower bound is nonnegative.

**Proposition 2**

Consider the following:

(53) $$\begin{array}{c} DK-\frac{{\left(DN\right)}^{2}}{4DM}\geq 0. \end{array}$$

**Proof**

Define two column-vectors as follows:

(54) $$\begin{array}{c} x≜TBC\left(0\right)=\left[\begin{array}{c} x_{1}\\ x_{2}\\ \vdots \\ x_{n} \end{array}\right]\in C^{n\times 1},\\ y≜\left(I+TA\right)C\left(0\right)=\left[\begin{array}{c} y_{1}\\ y_{2}\\ \vdots \\ y_{n} \end{array}\right]\in C^{n\times 1}, \end{array}$$

and *M*, *N*, and *K* can be rewritten as follows:

(55) $$\begin{array}{c} M=x\circ x^{*}=\left[\begin{array}{c} {\left|x_{1}\right|}^{2}\\ {\left|x_{2}\right|}^{2}\\ \vdots \\ {\left|x_{n}\right|}^{2} \end{array}\right],\\ N=x\circ y^{*}+y\circ x^{*}=2ℜ\left(x\circ y^{*}\right)=2\left[\begin{array}{c} ℜ\left(x_{1}y_{1}^{*}\right)\\ ℜ\left(x_{2}y_{2}^{*}\right)\\ \vdots \\ ℜ\left(x_{n}y_{n}^{*}\right) \end{array}\right],\\ K=y\circ y^{*}=\left[\begin{array}{c} {\left|y_{1}\right|}^{2}\\ {\left|y_{2}\right|}^{2}\\ \vdots \\ {\left|y_{n}\right|}^{2} \end{array}\right]. \end{array}$$

We can do the following calculation:

(56) (DM)(DK)−(DN)24 =(∑i=1ndi|xi|2)(∑i=1ndi|yi|2)−[∑i=1ndiℜ(xiyi∗)]2 =∑i=1n∑j=1ndi|xi|2dj|yj|2  −∑i=1n∑j=1ndiℜ(xiyi∗)djℜ(xjyj∗) =∑i=1n∑j=1ndidj[|xiyj∗|2−ℜ(xiyi∗)ℜ(xjyj∗)].

For *i* = *j*,

(57) didj[|xiyj∗|2−ℜ(xiyi∗)ℜ(xjyj∗)] =di2[|xiyi∗|2−|ℜ(xiyi∗)|2]≥0.

For *i* ≠ *j*,

(58) didj[|xiyj∗|2−ℜ(xiyi∗)ℜ(xjyj∗)]  +djdi[|xjyi∗|2−ℜ(xjyj∗)ℜ(xiyi∗)] =didj[|xjyi∗|2+|xjyi∗|2−2ℜ(xiyi∗)ℜ(xjyj∗)] =didj[|xjyi∗|2−2|xiyj∗||xjyi∗|+|xjyi∗|2       +2|xiyj∗||xjyi∗|−2|ℜ(xiyi∗)||ℜ(xjyj∗)|       +2|ℜ(xiyi∗)||ℜ(xjyj∗)|−2ℜ(xiyi∗)ℜ(xjyj∗)] =didj{(|xiyj∗|−|xjyi∗|)2       +2[|xiyi∗||xjyj∗|−|ℜ(xiyi∗)||ℜ(xjyj∗)|]       +2[|ℜ(xiyi∗)ℜ(xjyj∗)|−ℜ(xiyi∗)ℜ(xjyj∗)]} ≥0.

So, (*DM*)(*DK*) − ((*DN*)^2^/4) ≥ 0, which implies *DK* − ((*DN*)^2^/4*DM*) ≥ 0.

Proposition 2 shows that *S*_*d*_(*T*) has a nonnegative lower bound, which will affect the selection of the target. When the lower bound is smaller than *S*_*d*_(0), the entropy can be reduced; otherwise, the entropy cannot be reduced in time *T*. However, it is possible to reduce the entropy after *T* using suitable control, which will be demonstrated by simulation in Section 6.1. This conclusion coincides with our common sense. Just as we know, it is always easy to make a system disordered, but it is not always easy to make a system ordered. To investigate when the entropy cannot be reduced, we can calculate the gap between the lower bound and *S*_*d*_(0) as follows:

(59) Sd(0)−[DK−(DN)24DM] =1DM{(DN)24−DM[DK−Sd(0)]} =1D[TBC(0)∘TB∗C∗(0)]  ×{14{2Dℜ[TBC(0)∘(I+TA∗)C∗(0)]}2    −D[TBC(0)∘TB∗C∗(0)]    ×{D[(I+TA)C(0)∘(I+TA∗)C∗(0)]       −D[C(0)∘C∗(0)]}} =1T2D[BC(0)∘B∗C∗(0)]  ×{{Dℜ[TBC(0)∘(I+TA∗)C∗(0)]}2     −D[TBC(0)∘TB∗C∗(0)]     ×D[TAC(0)∘C∗(0)+TC(0)∘A∗C∗(0)            + T2AC(0)∘A∗C∗(0)]} =T2T2D[BC(0)∘B∗C∗(0)]  ×{{Dℜ[BC(0)∘(I+TA∗)C∗(0)]}2     −D[BC(0)∘B∗C∗(0)]     ×D{2Tℜ[AC(0)∘C∗(0)]        + T2AC(0)∘A∗C∗(0)}} =1D[BC(0)∘B∗C∗(0)]  ×{{Dℜ[BC(0)∘C∗(0)]          + TDℜ[BC(0)∘A∗C∗(0)]}2     −2TD[BC(0)∘B∗C∗(0)]Dℜ[AC(0)∘C∗(0)]     − T2D[BC(0)∘B∗C∗(0)]D[AC(0)∘A∗C∗(0)]}.

Let

(60) $$\begin{array}{c} S_{d}\left(0\right)-\left[DK-\frac{{\left(DN\right)}^{2}}{4DM}\right]=\frac{aT^{2}+bT+c}{D\left[BC\left(0\right)\circ B^{*}C^{*}\left(0\right)\right]}, \end{array}$$

where

(61) a={Dℜ[BC(0)∘A∗C∗(0)]}2−D[BC(0)∘B∗C∗(0)]D[AC(0)∘A∗C∗(0)],b=2Dℜ[BC(0)∘C∗(0)]Dℜ[BC(0)∘A∗C∗(0)]−2D[BC(0)∘B∗C∗(0)]Dℜ[AC(0)∘C∗(0)],c={Dℜ[BC(0)∘C∗(0)]}2.

From *D*[*BC*(0)∘B*C*(0)] ≥ 0, we know that the entropy cannot be reduced when lim_*T*→0_(*aT* ^2^ + *bT* + *c*) ≤ 0. The following proposition shows that *a* ≤ 0.

**Proposition 3**

Consider the following:

(62) a={Dℜ[BC(0)∘A∗C∗(0)]}2−D[BC(0)∘B∗C∗(0)]D[AC(0)∘A∗C∗(0)]≤0.

**Proof**

Assume

(63) $$\begin{array}{c} BC\left(0\right)=\left[\begin{array}{c} p_{1}\\ p_{2}\\ \vdots \\ p_{n} \end{array}\right]\in C^{n\times 1},\;\;AC\left(0\right)=\left[\begin{array}{c} q_{1}\\ q_{2}\\ \vdots \\ q_{n} \end{array}\right]\in C^{n\times 1}, \end{array}$$

and *a* can be rewritten as follows:

(64) a=[∑j=1ndiℜ(piqi∗)]2−(∑i=1ndi|pi|2)(∑i=1ndi|qi|2)=∑i=1n∑j=1ndiℜ(piqi∗)djℜ(pjqj∗)−∑i=1n∑j=1ndi|pi|2dj|qj|2=∑i=1n∑j=1ndidj[ℜ(piqi∗)ℜ(pjqj∗)−|pi|2|qj|2].

For *i* = *j*,

(65) didj[ℜ(piqi∗)ℜ(pjqj∗)−|pi|2|qj|2] =di2[|ℜ(piqi∗)|2−|piqi∗|2]≤0.

For *i* ≠ *j*,

(66) didj[ℜ(piqi∗)ℜ(pjqj∗)−|pi|2|qj|2]  +djdi[ℜ(pjqj∗)ℜ(piqi∗)−|pj|2|qi|2] =didj[2ℜ(piqi∗)ℜ(pjqj∗)−|piqj∗|2−|pjqi∗|2] ≤didj[2|ℜ(piqi∗)||ℜ(pjqj∗)|−|piqj∗|2−|pjqi∗|2]   ≤didj[2|piqi∗||pjqj∗|−|piqj∗|2−|pjqi∗|2] =−didj(|piqj∗|−|pjqi∗|)2≤0.

We can conclude that *a* ≤ 0.

Based on Proposition 3, we can find out under what conditions the entropy cannot be reduced, which is shown in Theorem 4.

**Theorem 4**

The entropy cannot be reduced in very small time *T* when

(67) $$\begin{array}{c} Dℜ\left[AC\left(0\right)\circ C^{*}\left(0\right)\right]\geq 0,\;\;Dℜ\left[BC\left(0\right)\circ C^{*}\left(0\right)\right]=0. \end{array}$$

**Proof**

**Figure 1:**
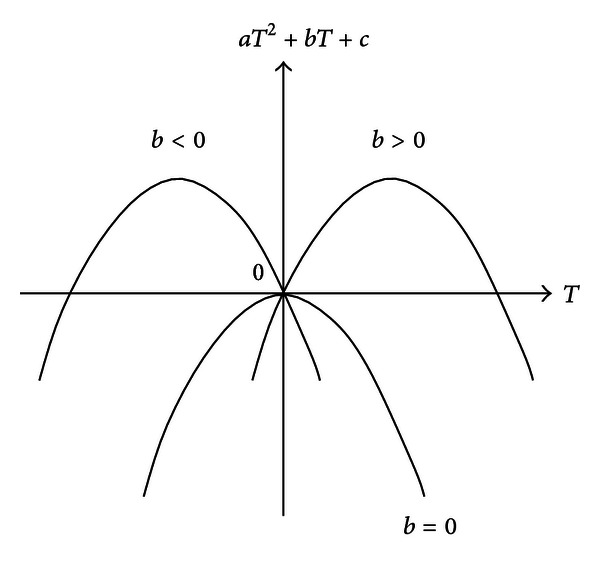
The relationship between *aT* ^2^ + *bT* + *c* and *T*.

From (61) we know *c* ≥ 0. When *c* > 0, it is clear that lim_*T*→0_(*aT* ^2^ + *bT* + *c*) > 0, which means that the entropy can be reduced. When *c* = 0 and *a* = 0, lim_*T*→0_(*aT* ^2^ + *bT* + *c*) ≤ 0 is true only when *b* ≤ 0. When *c* = 0 and *a* < 0, from Figure 1, we can see that lim_*T*→0_(*aT* ^2^ + *bT* + *c*) ≤ 0 only holds when *b* ≤ 0.

So the conditions under which the entropy can only be reduced are *c* = 0 and *b* ≤ 0. From (61) we know *c* = 0 implies *Dℜ*[*BC*(0)∘C*(0)] = 0 which yields *b* = −2*D*[*BC*(0)∘B*C*(0)]*Dℜ*[*AC*(0)∘C*(0)]. From *Dℜ*[*BC*(0)∘B*C*(0) ≥ 0, we know that, to get *b* ≤ 0, we only need *Dℜ*[*AC*(0)∘C*(0)] ≥ 0. We can conclude the result in Theorem 4.

Theorem 4 gives the conditions under which the entropy cannot be reduced. During practical control process, we do not need to do the calculations in (67) at every step. This is because if one wants to reduce the entropy when it cannot be reduced, the selection of *S*_*d*_(*T*) must be smaller than its lower bound, which makes (*DN*)^2^ − 4*DM*[*DK* − *S*_*d*_(*T*)] < 0, and the controller (51) will be unsolvable. Another question is that, even when the entropy can be reduced, we cannot reduce it below the lower bound in one time step *T*. In order to reduce it below the lower bound, multistep tracking can be adopted since Theorem 1 only holds for small change of the probability. Although fast probability change may lead to fast entropy decreasing, it cannot be tracked and approximated with Theorem 1.

**Figure 2:**
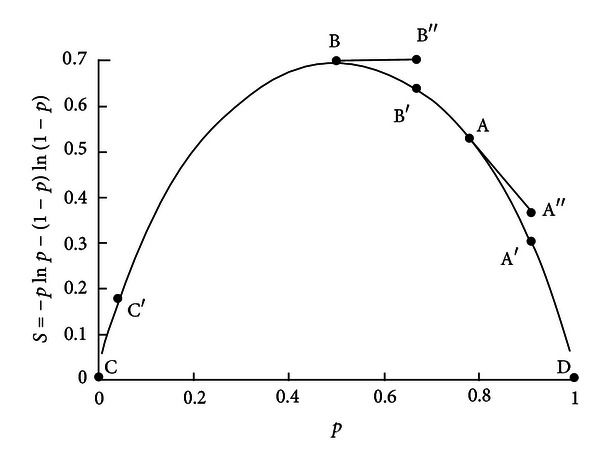
The relationship between entropy and probability for two-level quantum systems.

 We can show the essence of the algorithm in Figure 2 based on two-level quantum systems. Assuming |*c*_1_(*t*)|^2^ = *p*, the entropy of two-level systems becomes *S* = −*p*ln⁡*p* − (1 − *p*)ln⁡(1 − *p*). The relationship between entropy and probability can be depicted in Figure 2.

For arbitrary point *A*, when the system goes from *A* to *A*′, the probability change can be denoted as Δ*p* = *p*_*A*′_ − *p*_*A*_. If we denote the entropy at *A*′ as *S*_*A*′_ and approximate it with *S*_*A*′′_, the approximation error *e* = *S*_*A*′_ − S_*A*′′_ should satisfy lim_Δ*p*→0_(*e*/Δ*p*) = 0. Obviously there will be some delay in such an approximation. Hence, in Section 6.2, we will use prediction to achieve more accurate tracking.

 It should be noted that for the entropy's maximum point *B* and minimum points *C* and *D*, our algorithm cannot be applied. For point *B*, we have d*S*/d*p* = 0 and *S*_*B*′′_ = *S*_*B*_, so the entropy will not change under the approximation of *S*_*B*′′_. For quantum systems with *n* levels, the maximum point satisfies |*c*_*i*_(0)|^2^ = (1/*n*)  (for all *i*), so we can get *S*_*d*_(*T*) = −∑_*i*=1_^*n*^|*c*_*i*_(*T*)|^2^ln⁡⁡|*c*_*i*_(0)|^2^ = −∑_*i*=1_^*n*^|*c*_*i*_(*T*)|^2^ln⁡(1/*n*) = ln⁡*n* · ∑_*i*=1_^*n*^|*c*_*i*_(*T*)|^2^ = ln⁡*n* = *S*_*d*_(0), which will not change the entropy either. For point *C* we have d*S*/d*p* = +*∞*, lim_Δ*p*→0_(*e*/Δ*p*) = −*∞*, and for point *D* we have d*S*/d*p* = −*∞*, lim_Δ*p*→0_(*e*/Δ*p*) = +*∞*, which make the algorithm unfeasible. For three-level systems, when the entropy is at its minimum, one of |*c*_*i*_(*t*)|^2^  (*i* = 1,2, 3) must be 1, and the others must be 0. Assume the vector [|*c*_1_(*t*)|^2^, |*c*_2_(*t*)|^2^, |*c*_3_(*t*)|^2^] goes from [1,0, 0]  to [1 − Δ_1_ − Δ_2_, Δ_1_, Δ_2_]  (Δ_1_ > 0, Δ_2_ > 0). Such a process is equivalent to [1,0, 0]→[1 − Δ_1_ − Δ_2_, Δ_1_ + Δ_2_, 0]→[1 − Δ_1_ − Δ_2_, Δ_1_, Δ_2_]. If we denote the errors in each change as *e*_1_ and *e*_2_, we have lim_Δ_1_+Δ_2_→0_(*e*_1_/2(Δ_1_ + Δ_2_)) = lim_Δ_2_→0_(*e*_2_/2Δ_2_) = −*∞*, which leads to the following:

(68) lim⁡Δ1+Δ2→0⁡e1+e22(Δ1+Δ2) =lim⁡Δ1+Δ2→0⁡[e12(Δ1+Δ2)+e22Δ2Δ2Δ1+Δ2] =lim⁡Δ1+Δ2→0⁡e12(Δ1+Δ2)  +Δ2Δ1+Δ2lim⁡Δ1+Δ2→0⁡e22Δ2   =−∞+Δ2Δ1+Δ2(−∞)=−∞.

The same conclusion holds for quantum systems with more than three levels.

## 6. Simulation Examples

In order to illustrate the effectiveness of our algorithm, we present simulation examples on both two-level and three-level quantum systems.

### 6.1. Simulation on a Two-Level System

Consider the system

(69) $$\begin{array}{c} \left[\begin{array}{c} {\dot{c}}_{1}\left(t\right)\\ {\dot{c}}_{2}\left(t\right) \end{array}\right]=\left\{\left[\begin{array}{cc} -\iota & 0\\ 0 & \iota \end{array}\right]+\left[\begin{array}{cc} 0 & -1\\ 1 & 0 \end{array}\right]U\left(t\right)\right\}\left[\begin{array}{c} c_{1}\left(t\right)\\ c_{2}\left(t\right) \end{array}\right]. \end{array}$$

It is easy to verify that *DM* = −*T* ^2^[|*c*_1_(0)|^2^ln⁡|*c*_2_(0)|^2^ + |*c*_2_(0)|^2^ln⁡|*c*_1_(0)|^2^] > 0, so we should always use (51) to calculate *U*(0). It is easy to obtain the following:

(70) $$\begin{array}{c} Dℜ\left[AC\left(0\right)\circ C^{*}\left(0\right)\right]\equiv 0,\\ Dℜ\left[BC\left(0\right)\circ C^{*}\left(0\right)\right]=ℜ\left[c_{1}\left(0\right)c_{2}^{*}\left(0\right)\right]\ln\frac{{\left|c_{2}\left(0\right)\right|}^{2}}{{\left|c_{1}\left(0\right)\right|}^{2}}. \end{array}$$

If the entropy is not at its maximum or minimum, we have ln⁡(|*c*_2_(0)|^2^/|*c*_1_(0)|^2^) ≠ 0 or ±*∞*, and, from Theorem 4, we know that the condition under which the entropy cannot be reduced is as follows:

(71) $$\begin{array}{c} ℜ\left[c_{1}\left(0\right)c_{2}^{*}\left(0\right)\right]=0. \end{array}$$

**Figure 3:**
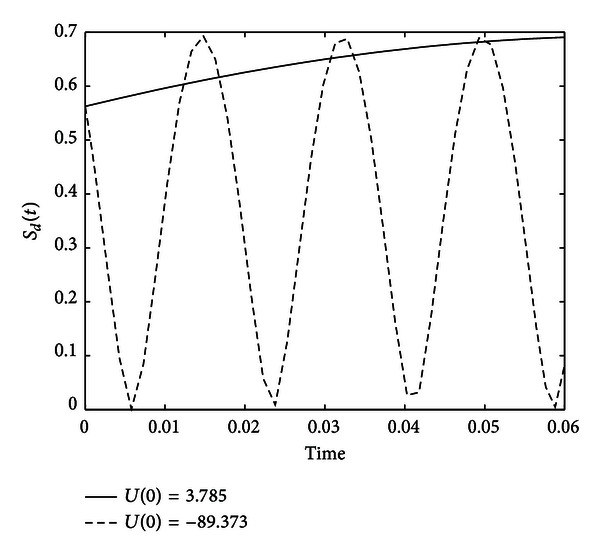
Evolutions of the entropy for system (69) with initial state $C\left(0\right)={\left[\sqrt{3}/2,1/2\right]}^{T}$ under controller *U*(0) = 3.785 and −89.373.

For initial state $C(0)={\left[\sqrt{3}/2,1/2\right]}^{T}$ which does not satisfy (71), we have *S*_*d*_(0) = 0.562. If the entropy is desired to increase to *S*_*d*_(*T*) = 0.6 at time *T* = 0.01, the controller (51) can be calculated as *U*(0) = 3.875 or −89.373. Simulations of the entropy for system (69) with initial state $C(0)={\left[\sqrt{3}/2,1/2\right]}^{T}$ under controller *U*(0) = 3.785 and −89.373 are shown in Figure 3.

**Figure 4:**
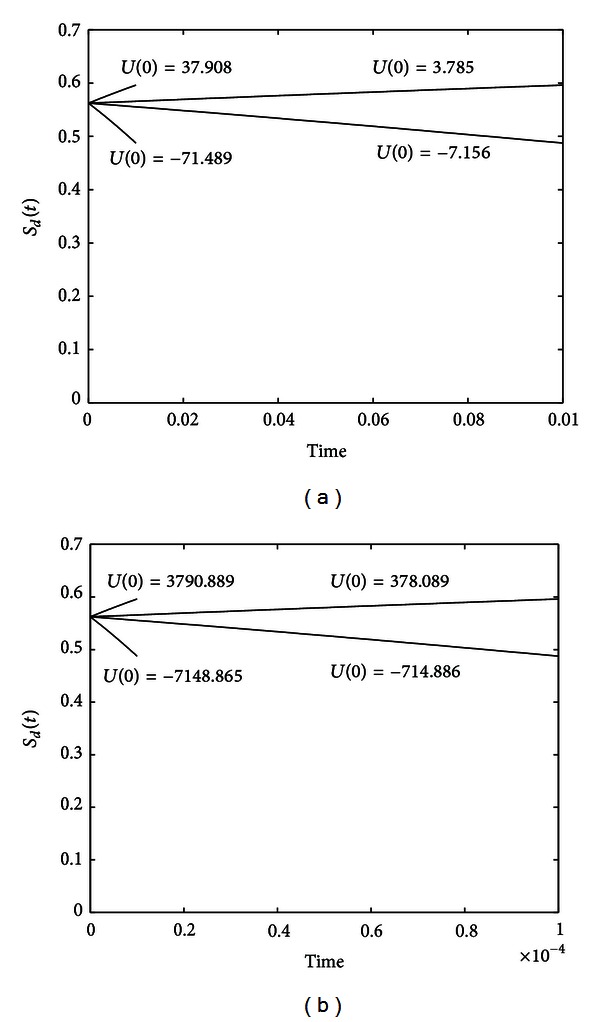
Evolutions of the entropy for system (69) with initial state $C\left(0\right)={\left[\sqrt{3}/2,1/2\right]}^{T}$, when *S*_*d*_(*T*) = 0.6 and 0.5, *T* = 0.01, 0.001, 0.0001, and 0.00001.

From Figure 3 we can see that both controllers can achieve the goal. While *U*(0) = −89.373 can make the probability change very fast. This does not satisfy the premise of Theorem 1, thus the entropy cannot be accurately approximated. From (69) we can see when *U*(0) = 0, there will be no change in the probability distribution and the entropy. Since larger |*U*(0)| leads to faster entropy change with oscillation, we just choose the controller with small modulus. Hence, in (51), when *DN* > 0 we choose plus; otherwise we choose minus. For *S*_*d*_(*T*) = 0.6 and 0.5, when *T* = 0.01, 0.001, 0.0001, and 0.00001, the simulations are shown in Figure 4. 

We can see the entropy can be driven to its destination at any prespecified time, which can be accomplished very quickly in one step. When the entropy has reached its target, from (23), we know that we can just apply *U*(*t*) = 0 to maintain the entropy unchanged for diagonal *A*. Here, the change of entropy in one step cannot be very large because Theorem 1 only holds for small change of the probability. In Section 6.2, we will show that multiple step tracking can be used to deal with large entropy change.

**Figure 5:**
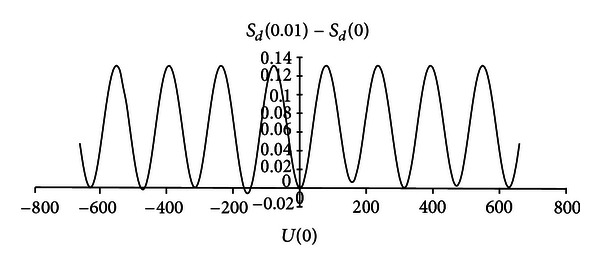
Change of the entropy with respect to *S*_*d*_(0) at *T* = 0.01 under different *U*(0) for system (69) with $C\left(0\right)={\left[\sqrt{3}/2,\iota /2\right]}^{T}$.

 For initial state $C(0)={\left[\sqrt{3}/2,\iota /2\right]}^{T}$, which satisfies (71), the entropy cannot be reduced in very small time *T* with constant *U*(0). This can be seen in Figure 5 which shows the change of entropy with respect to *S*_*d*_(0) at *T* = 0.01 under different *U*(0).

**Figure 6:**
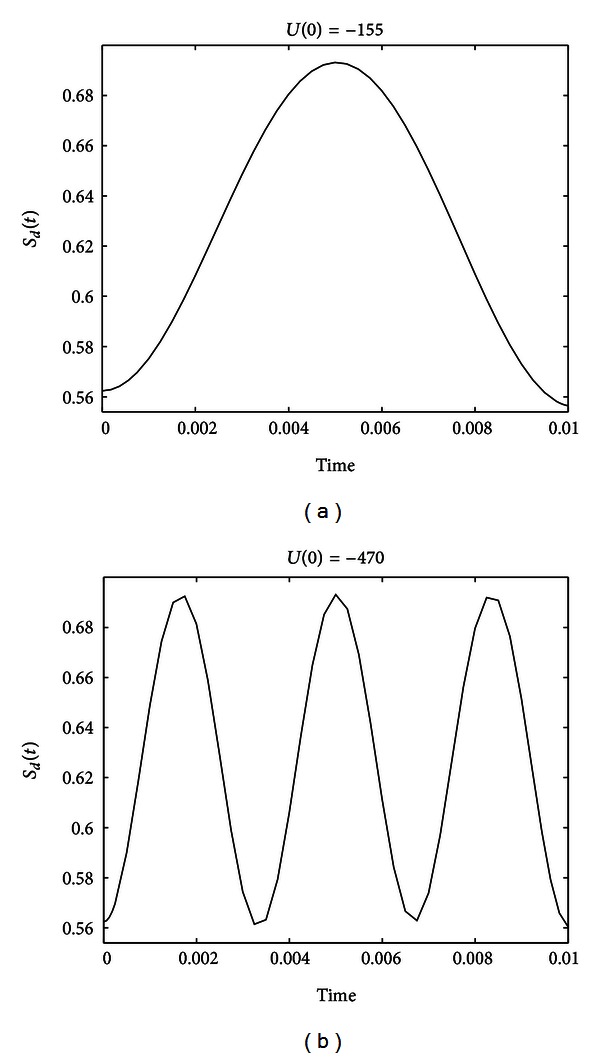
Evolutions of the entropy under *U*(0) = −155 and −470 for system (69) with initial state $C\left(0\right)={\left[\sqrt{3}/2,\iota /2\right]}^{T}$.

From Figure 5, we can see that, no matter how large *U*(0) is, the entropy at *T* = 0.01 is almost always larger than *S*_*d*_(0) except when *U*(0) = −155 and −470. The evolutions of the entropy under *U*(0) = −155 and −470 are shown in Figure 6. 

From Figure 6, we can find that the entropy cannot be reduced at the beginning, but can be reduced later, which coincides with Theorem 4.

### 6.2. Simulation on a Three-Level System

 Consider the following system:

(72) $$\begin{array}{c} \left[\begin{array}{c} {\dot{c}}_{1}\left(t\right)\\ {\dot{c}}_{2}\left(t\right)\\ {\dot{c}}_{3}\left(t\right) \end{array}\right]=\left\{\left[\begin{array}{ccc} \iota & -\iota & 0\\ -\iota & 0 & 0\\ 0 & 0 & -\iota \end{array}\right]+\left[\begin{array}{ccc} 0 & 0 & -\iota \\ 0 & 0 & 0\\ -\iota & 0 & 0 \end{array}\right]U\left(t\right)\right\}\left[\begin{array}{c} c_{1}\left(t\right)\\ c_{2}\left(t\right)\\ c_{3}\left(t\right) \end{array}\right]. \end{array}$$

Since *DM* = −*T* ^2^[|*c*_1_(0)|^2^ln⁡|*c*_3_(0)|^2^ + |*c*_3_(0)|^2^ln⁡|*c*_1_(0)|^2^] > 0, we should always use (51) to calculate *U*(0). The conditions under which the entropy cannot be reduced are as follows:

(73) $$\begin{array}{c} Dℜ\left[AC\left(0\right)\circ C^{*}\left(0\right)\right]=ℑ\left[c_{1}\left(0\right)c_{2}^{*}\left(0\right)\right]\ln\frac{{\left|c_{1}\left(0\right)\right|}^{2}}{{\left|c_{2}\left(0\right)\right|}^{2}}\geq 0,\\ Dℜ\left[BC\left(0\right)\circ C^{*}\left(0\right)\right]=ℑ\left[c_{1}\left(0\right)c_{3}^{*}\left(0\right)\right]\ln\frac{{\left|c_{1}\left(0\right)\right|}^{2}}{{\left|c_{3}\left(0\right)\right|}^{2}}=0. \end{array}$$

Assuming $C(0)={\left[\sqrt{6}/6,\sqrt{3}/3,\sqrt{2}\iota /2\right]}^{T}$, which does not satisfy the conditions, we have *S*_*d*_(0) = 1.011. In seven steps, we expect that the entropy changes as follows: (a) increases to 1.05; (b) remains unchanged; (c) increases to 1.1; (d) remains unchanged; (e) decreases to 1.05; (f) increases to 1.1; (g) remains unchanged. The controller can be calculated as follows, where 1(*t* − *t*_0_) denotes the unit step function starting from *t*_0_.

Consider the following:

(74) U(t)=5.582−4.385·1(t−0.01)+7.603·1(t−0.02)−3.926·1(t−0.03)−14.667·1(t−0.04)+17.137·1(t−0.05)−1.452·1(t−0.06).

**Figure 7:**
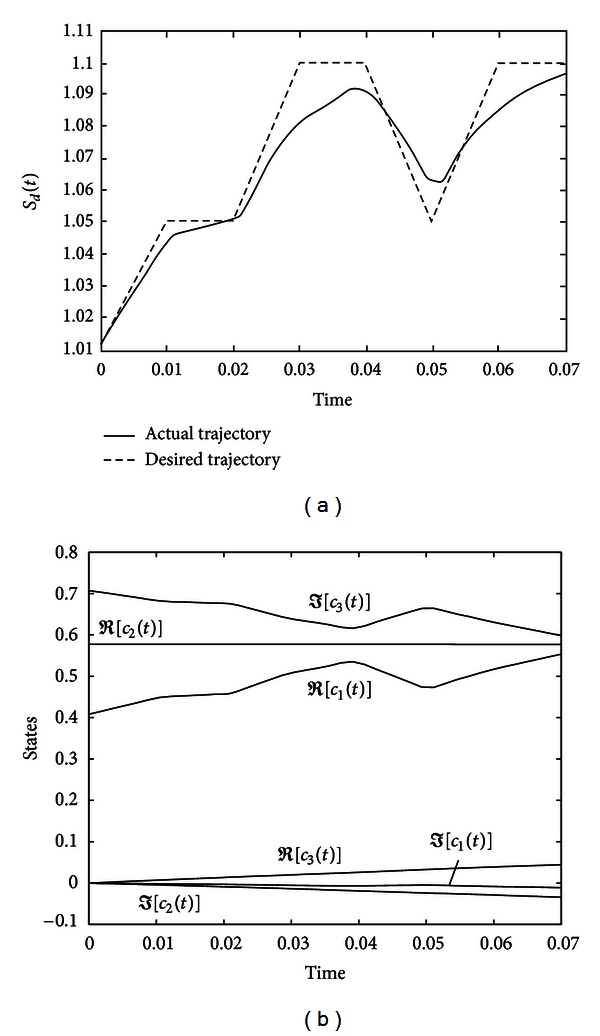
Evolutions of the entropy and states for system (72) under controller (74).

The evolutions of the entropy and the quantum states are shown in Figure 7.

**Figure 8:**
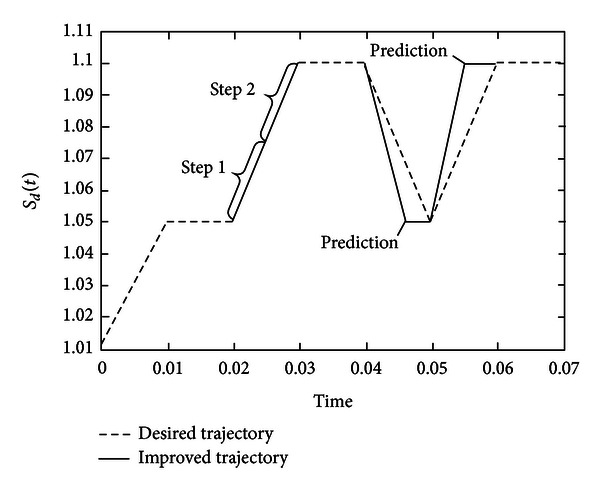
Improved control strategy with division and prediction.

In order to overcome the delays, we can divide one step into halves and use predictions, which can be shown in Figure 8.

The time interval (0.02,0.03) is divided into two steps, and for each step the controller is calculated separately. At times 0.045 and 0.055 we use half step predictions which can lead to more accurate control. The improved controller is as follows:

(75) U(t)=5.582−4.385·1(t−0.01)+8.204·1(t−0.02)+4.153·1(t−0.025)−9.645·1(t−0.03)−17.717·1(t−0.04)+30.549·1(t−0.05)−13.287·1(t−0.06).

**Figure 9:**
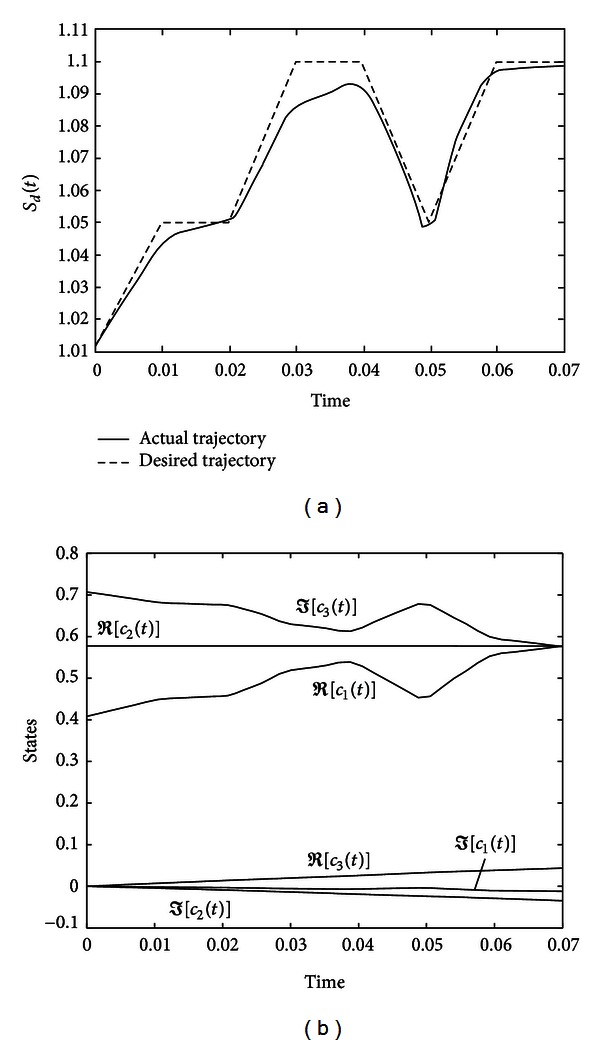
Evolutions of the entropy and states for system (72) under improved controller (75).

The simulations are shown in Figure 9.

## 7. Conclusion

This paper proposes a new quantum control method which controls the Shannon entropy of quantum systems. Simulation examples evidenced the effectiveness of the method. A strength of our method is that it provides a direct control algorithm for discrete quantum entropy, rather than the indirect one via PDF control. Our method provides a universal tool for entropy control, which can also contribute to classical information theory. Some immediate extensions of the method include quantum sliding-mode control and coherent control. The extension of the methods to the mixed state case deserves our future research. The applications in correlation energy and biological control are also of keen interests and currently being pursued.
